# Association of cognitive impairment and diabetes on survival in Chinese older people with hypertension: a 10-year prospective study

**DOI:** 10.1186/s12877-023-04214-4

**Published:** 2023-09-21

**Authors:** Shuang Cai, Bokai Cheng, Kailiang Li, Yun Li, Anhang Zhang, Jin Sun, Yongkang Su, Man Li, Qiligeer Bao, Yan Zhang, Shouyuan Ma, Ping Zhu, Shuxia Wang

**Affiliations:** 1 Department of Geriatrics, The Second Medical Center & National Clinical Research Center for Geriatric Diseases, Chinese PLA General Hospital, Beijing, 100853 China; 2 Medical School of Chinese PLA, Beijing, 100853 China; 3 Department of Geriatric Cardiology, The Second Medical Center, Chinese PLA General Hospital, 28 Fuxing Road, Beijing, 100853 China; 4 Department of Emergency, The Fifth Medical Center, Chinese PLA General Hospital, Beijing, 100853 China; 5 Department of Outpatient, The First Medical Center, Chinese PLA General Hospital, Beijing, 100853 China

**Keywords:** Hypertension, Diabetes mellitus, Cognitive impairment, Comorbidities, All-cause mortality

## Abstract

**Background:**

Older people with hypertension may have more complex multisystem problems and a higher risk of morbidity and mortality. We aimed to examine the association of cognitive impairment (CI) and diabetes mellitus (DM) on all-cause mortality in the aged with hypertension (HTN).

**Methods:**

This is a prospective cohort study with a sample of 1017 older people with hypertension aged 60 years or older who completed baseline examinations in 2009–2010 and followed up for ten years in 2020. The endpoint was death from any cause. Subjects were categorized as HTN only, HTN + DM, HTN + CI, and HTN + DM + CI. Cox regression model was used to determine the association of comorbidities on all-cause mortality.

**Results:**

During the 10-year follow-up period, 196 deaths occurred. After adjusted for covariates, risk of death from any cause was significantly increased in the older people with increased comorbidities (P = 0.003). Compared with the HTN only, with HTN + CI, and HTN + DM + CI, the HRs (95% confidence intervals) for all-cause mortality were 1.61(1.13–2.30) and 1.79(1.07–2.99), respectively. In stratified analyses, the relationship between comorbidities level and the risk of all-cause mortality persisted.

**Conclusion:**

All-cause mortality risks increased with increasing the comorbidities. This study emphasizes the importance of comprehensive management of the older people with HTN in clinical practice and public health policy.

## Introduction

Comorbidities refer to a disease or other pathological process that occurs simultaneously with another, the extent to which two diseases occur together in the same population, which is characteristic of the older people [1, 2]. Comorbidities have a significant impact on the health of the older people and have a high incidence [3]. 65% of older adults have comorbidities, and 43% of older adults have three or more chronic conditions in the United States [4]. In Germany, 62% have three or more chronic problems [5]. Increased use of medical resources by older persons with comorbidities is common [6].

Hypertension, diabetes mellitus, and cognitive impairment are common problems in the older people, their prevalence increases with age, and they are important risk factors for cardiovascular morbidity and mortality [7–10]. These three situations often coexist. It is a management problem for the relevant practitioners. At present, in the health management of the older people, the management of chronic diseases is primarily single and fragmented. Multiple chronic problems are superimposed, and the focus of intervention is not prominent. They neglected geriatric problems and functional status, ignored the overall treatment of health problems, and lacked a unified plan and technology for managing comorbidities in the older people. Treatment guidelines for a single disease are often derived from studies in non-comorbid populations. Therefore, elucidating the extent of the cumulative association of the comorbidities of HTN on risk may contribute to the development of approaches to managing comorbidities in older adults. Our study was derived from the older people hypertensive samples from Wanshou Road, Beijing. The study investigated the relationship between the comorbidities in hypertension and all-cause mortality.

## Methods

### Study population

Our study program and sampling have been described previously [11–13]. In short, the subjects were all from a community cross-sectional survey of people aged 60 and over in Wanshou Road Community, Haidian District, Beijing, from September 2009 to June 2010. A total of 2,162 subjects (female: 60.1%) completed the survey. Among them, 1167 hypertensive subjects were identified and thoroughly examined. HTN was defined as having systolic blood pressure (SBP) 140 mmHg or greater, diastolic blood pressure (DBP) 90 mmHg or greater, or taking medication for hypertension [14]. DM refers to fasting blood glucose ≥ 7.0 mmol/L after fasting for more than 8 h or the 2-h plasma glucose value after a 75-g oral glucose tolerance test ≥ 11.1 mmol/L or blood glucose ≥ 11.1 mmol/L at any time or diabetes diagnosed by a doctor [15]. In this study, a mini-mental state examination (MMSE) score ≥ 27 was classified as positive cognitive function, while an MMSE score<27 was considered as CI [16]. Of 1167 hypertensive subjects, 150 participants were excluded for loss of follow-up. We included subjects with HTN only as a reference group (excluding those diagnosed with DM and/or CI) and divided them into the following four categories:

1. HTN only (reference group).
2. HTN + DM
3. HTN + CI
4. HTN + DM + CI

The study protocol was reviewed and approved by the ethical committees of the Chinese PLA General Hospital. The research procedures followed the ethically normative criteria. Written informed consent was acquired from all subjects.

### Outcomes

Details of the follow-up in this study have been described in previous studies [12, 17]. Briefly, the end point of this study was death from any cause. Follow-up ended in Dec.2020, and survival was defined as the number of months from recruitment to death or the end of observation (Dec. 31, 2020). Vital status information was obtained through telephone interviews with family members or other caregivers. The subjects’ identities were verified by information such as name, age, and sex.

### Data collection

The researchers assessed participants’ demographic characteristics, including demographic factors, medical history, and lifestyle, through face-to-face interviews using standard questionnaires. Lifestyle includes drinking and smoking. Alcohol consumption and smoking were considered dichotomous variables for never/former and current. Cognitive functions of all individuals were performed using the Chinese MMSE, which was adapted from the original English MMSE, adapted to China’s cultural background, and validated in previous studies based on the Chinese population [18]. Blood pressure was measured according to standardized protocols. Two blood pressure recordings (5-min intervals) were obtained from participants’ right arms in a sitting position after 30 min of rest. The blood pressure was measured using a sphygmomanometer, and the average of the two was used for analysis. Fasting blood samples were taken from all subjects in the morning (after fasting for at least 12 h). An automatic biochemical analyzer measured serum lipids, glucose, routine blood tests, and creatinine. All biochemical analyses were performed in the Chinese PLA General Hospital Department of Biochemistry.

### Statistical analysis

Descriptive analyses were used to report baseline characteristics of participants in four categories based on the level of multimorbidity. Continuous variables were reported as mean ± standard deviation (SD) and categorical variables as percentages. Subjects are categorized as follows: (1) HTN only (reference group), (2) HTN + DM, (3) HTN + CI, (4) HTN + DM + CI. Baseline characteristics between subjects in different groups were compared using the χ2 test and analysis of variance. Cox proportional hazards models were used for estimating the association between risk of all-cause mortality and comorbidities, estimating HRs and 95% confidence intervals. We developed three models which adjusted for potential confounders. Model 1: unadjusted; Model 2: adjusted for sex, age; Model 3: adjusted for age, sex, SBP, DBP, total cholesterol (TC), high-density lipoprotein (HDL-C), low-density lipoprotein cholesterol (LDL-C), triglycerides (TG), serum uric acid (SUA), Serum creatinine (Scr), smoking, alcohol drinking, coronary heart disease (CHD), stroke, dyslipidemia, activities of daily living(ADL) scores, and baseline medication: aspirin, calcium channel blockers (CCB), beta-blocker, angiotensin converting enzyme (ACE) inhibitor, angiotensin receptor blocker (ARB). Subgroup analyses were stratified by sex (male or female), and age (< 75 or ≥ 75 years). All statistical tests were 2-sided, with *P* < 0.05 considered statistically significant. All analyses were performed using the statistical software packages statistical package for the social sciences (SPSS) (version 26.0).

## Results

### Baseline characteristics

Among the 2162 samples, 1167 were older people with hypertension, 150 were lost to follow-up, and 1017 were finally included in the study.

Table 1 shows the baseline characteristics of the older people with HTN categorized according to their comorbidity status: a total of 523 subjects were diagnosed with HTN only (51.4%), 154 (15.1%) had HTN + DM, 263 (25.9%) had HTN + CI, and 77 (7.6%) had HTN + DM + CI. Compared with HTN only, HTN + DM + CI tend to have higher age, SBP, FPG, a higher prevalence of CHD, stroke, dyslipidemia, and lower HDL-C and ADL scores. There were no significant differences in DBP, TG, LDL-C, Scr, smoking status, drinking status, and application of beta-blockers.

**Table 1:** Baseline demographics by combinations of comorbidities in older people with hypertension

Characteristics	HTN only	HTN + DM	HTN + CI	HTN + DM + CI	*P*-value
N	523	154	263	77	
Age (years)	70.71 ± 6.76	70.02 ± 6.03	74.23 ± 6.32	73.92 ± 4.61	< 0.001
Males (%)	222(42.4)	61(39.6)	78(29.7)	23(29.9)	0.002
SBP (mmHg)	140.05 ± 16.49	143.18 ± 17.81	145.45 ± 21.55	147.08 ± 22.33	< 0.001
DBP (mmHg)	77.91 ± 9.22	78.55 ± 9.65	78.63 ± 10.50	78.83 ± 11.53	0.698
FPG (mmol/L)	5.72 ± 0.92	7.18 ± 1.70	5.79 ± 1.07	8.07 ± 2.81	< 0.001
TC (mmol/L)	5.25 ± 1.01	5.06 ± 1.05	5.37 ± 1.03	5.15 ± 1.00	0.023
TG (mmol/L)	1.74 ± 0.88	1.74 ± 0.89	1.70 ± 0.82	1.93 ± 1.23	0.284
HDL-C (mmol/L)	1.39 ± 0.39	1.32 ± 0.35	1.43 ± 0.36	1.27 ± 0.30	0.001
LDL-C (mmol/L)	3.25 ± 0.87	3.11 ± 0.88	3.30 ± 0.86	3.14 ± 0.76	0.132
SCr (µmol/L)	76.04 ± 23.59	72.60 ± 20.42	75.36 ± 28.76	74.22 ± 24.64	0.487
SUA(µmol/L)	325.88 ± 94.50	322.24 ± 83.39	313.25 ± 93.64	296.35 ± 84.96	0.036
Smokers (%)	144(27.5)	46(29.9)	92(35.1)	25(32.5)	0.175
Drinkers (%)	131(25.0)	33(21.4)	57(21.7)	14(18.2)	0.439
CHD, n (%)	149(28.5)	62(40.3)	73(27.8)	28(36.4)	0.018
Stroke, n (%)	69(13.2)	29(18.8)	54(20.6)	21(27.3)	0.003
Dyslipidemia, n (%)	172(33.0)	67(43.8)	54(20.6)	30(39.0)	< 0.001
ADL scores, n (%)	99.50 ± 3.50	99.48 ± 4.25	97.68 ± 9.73	96.17 ± 8.81	< 0.001
Baseline medication, (%)					
Aspirin	150(28.8)	61(39.9)	71(27.3)	29(37.7)	0.018
CCB	321(61.6)	102(66.7)	140(53.4)	47(61.8)	0.042
Beta-blocker	75(14.4)	22(14.4)	35(13.4)	12(15.8)	0.955
ACE inhibitor	62(12.0)	19(12.3)	14(5.3)	8(10.8)	0.026
ARB	73(14.0)	34(22.1)	22(8.5)	13(17.1)	0.001

Abbreviations: HTN, hypertension; CI, Cognitive Impairment; SBP, systolic Blood pressure; DBP, diastolic blood pressure; FPG, fasting plasma glucose; TC, total cholesterol; HDL-C, high-density lipoprotein; LDL-C, low-density lipoprotein cholesterol; TG, Triglycerides; SUA, serum uric acid; Scr, Serum creatinine; CHD, Coronary heart disease; ADL, activities of daily living; CCB, calcium channel blockers; ACE, angiotensin converting enzyme; ARB, angiotensin receptor blocker

### Association between comorbidities and all-cause mortality

**Fig. 1:**
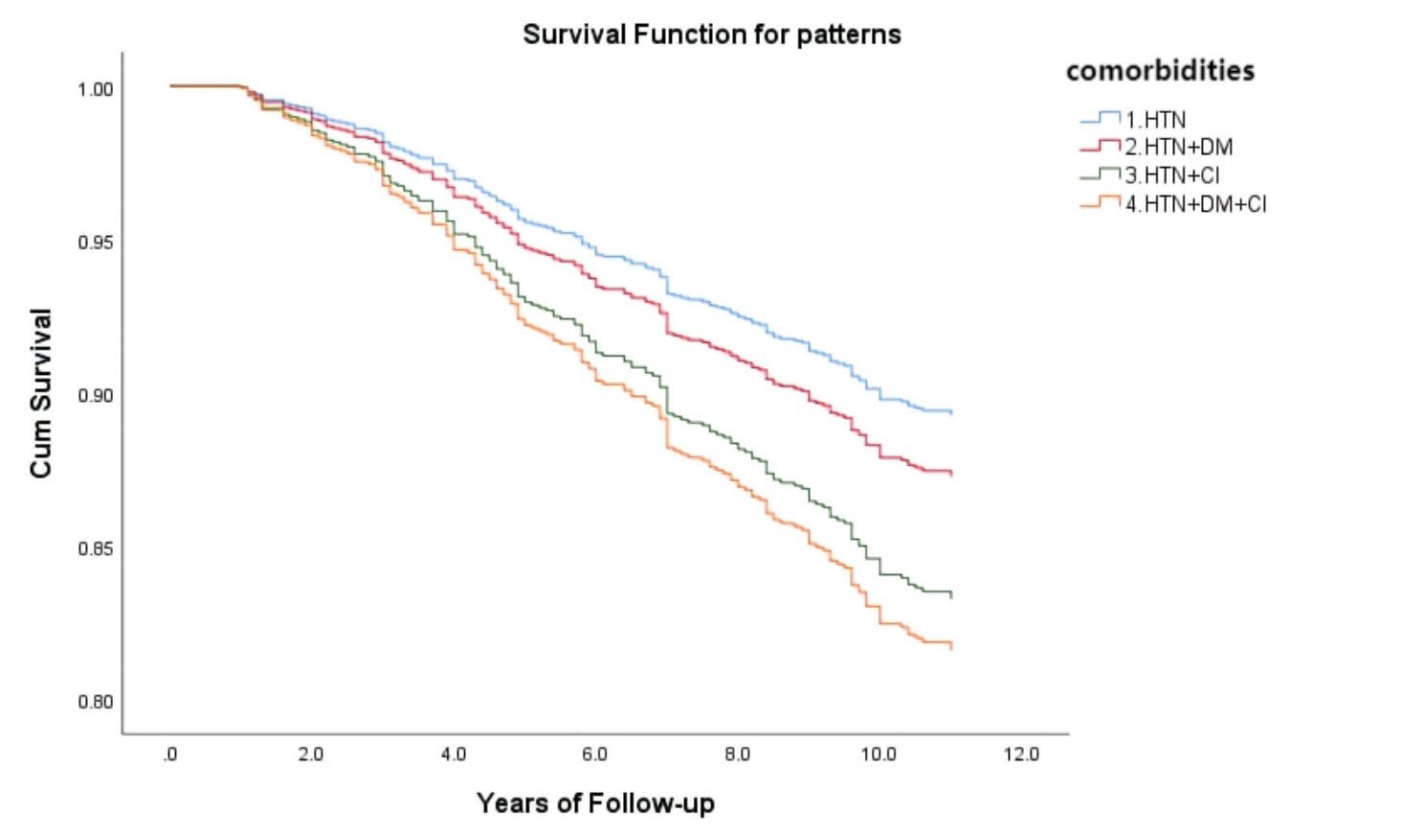
Cox proportional hazards after adjusted for age, sex, SBP, DBP, TC, HDL-C, LDL-C, TG, SUA, SCr, smoking, alcohol drinking, CHD, stroke, dyslipidemia, ADL scores, and baseline medication (aspirin, CCB, beta-blocker, ACE inhibitor, ARB)

Among 1017 participants (37.8% males), the median follow-up time was 10.8 years (1.0 to 11.3 years), and 196 deaths occurred. The cumulative incidence of death during follow-up was 13.9%. Figure 1; Table 2 show the association of comorbidities on all-cause mortality. For all models, no significant difference in HTN + DM. Univariate analysis (model 1), after adjustment for age and sex (model 2), and further for SBP, DBP, TC, HDL-C, LDL-C, TG, SUA, Scr, smoking, alcohol drinking, CHD, stroke, dyslipidemia, ADL scores, and baseline medication (aspirin, CCB, beta-blocker, ACE inhibitor, ARB) (model 3), the HRs for all-cause mortality remained progressively increased across HTN + CI and HTN + DM + CI. Specifically, compared with HTN only, HTN + CI increased the probability of all-cause mortality (HR 1.61, 95% confidence interval 1.13–2.30), as did the HTN + DM + CI (HR 1.79, 95%confidence interval 1.07–2.99).

**Table 2:** Cox regression analyses for the association of comorbidities on all-cause mortality

HR (95% confidence intervals)						
	Model 1	*P*-value	Model 2	*P*-value	Model 3	*P*-value
HTN only	1 (References)		1 (References)		1 (References)	
HTN + DM	1.03(0.66–1.64)	0.902	1.18(0.74–1.88)	0.501	1.20(0.74–1.94)	0.462
HTN + CI	2.12(1.54–2.93)	< 0.001	1.66(1.20–2.31)	0.002	1.61(1.13–2.30)	0.009
HTN + DM + CI	2.64(1.69–4.12)	< 0.001	1.94(1.24–3.04)	0.004	1.79(1.07–2.99)	0.026
*P* for trend	< 0.001		< 0.001		0.003	

Values are HRs (95% confidence intervals)

Abbreviation: HR, hazard ratio; HTN, hypertension; CI, Cognitive Impairment

Model 1: Unadjusted;

Model 2: Adjusted for sex, age;

Model 3: Adjusted for age, sex, SBP, DBP, TC, HDL-C, LDL-C, TG, SUA, SCr, smoking, alcohol drinking, CHD, stroke, dyslipidemia, ADL scores, and baseline medication (aspirin, CCB, beta-blocker, ACE inhibitor, ARB)

### Subgroup analyses for association between comorbidities and all-cause mortality

**Fig. 2:**
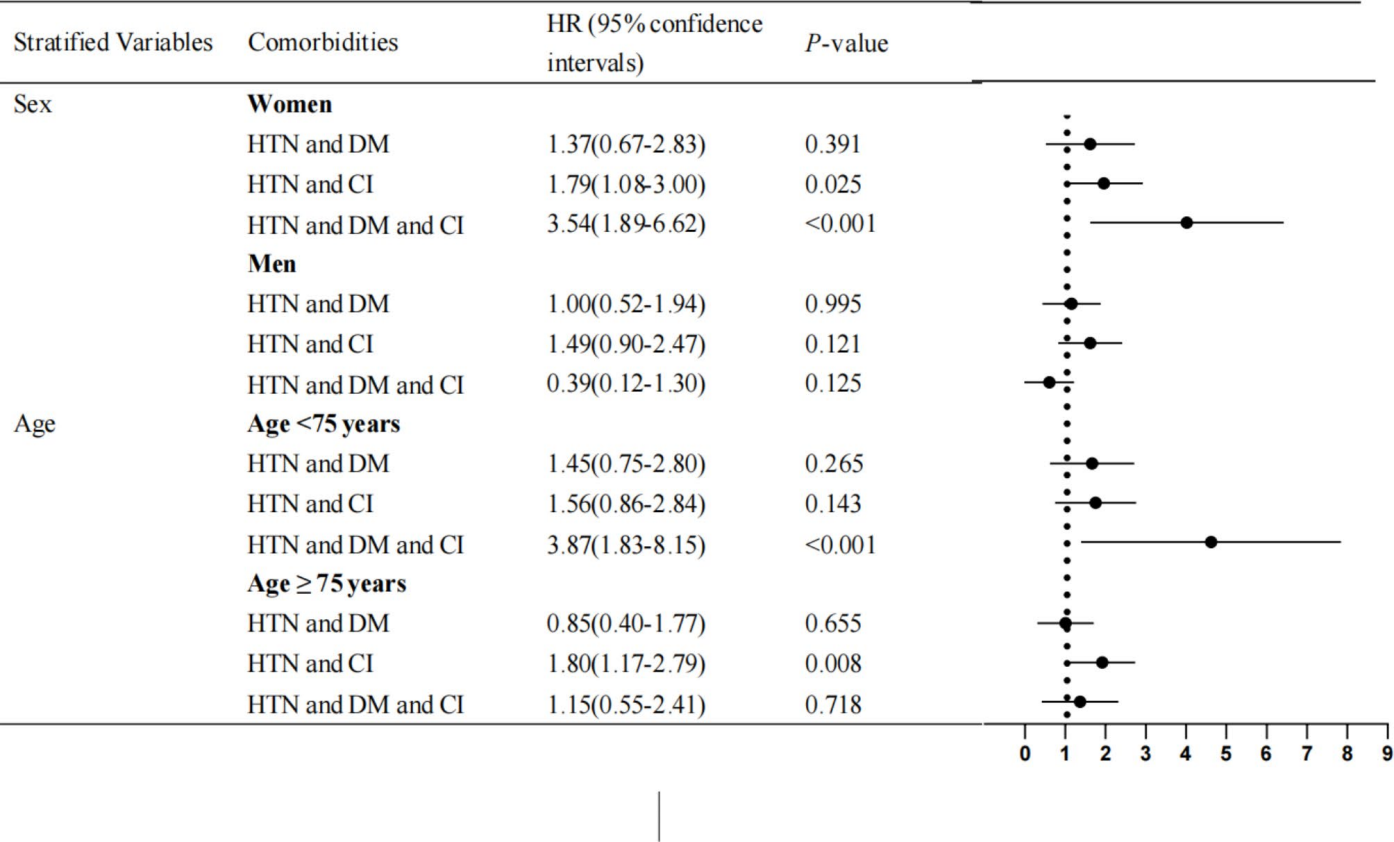
Subgroup analyses for association between comorbidities and all-cause mortality

We conducted a stratification analysis of the relationship between comorbidities and all-cause mortality. As shown in Fig. 2, in female stratification, the HTN + CI and HTN + DM + CI were significantly associated with increased all-cause mortality risk after adjustment for several covariates. However, the association was not significant in men. For<75 years, association with comorbidities was significant in HTN + DM + CI patients. For ≥ 75 years, the association between HTN + CI and all-cause mortality was significant.

## Discussion

The present study examined the association between comorbidities and all-cause mortality of HTN in the older people. To explore the relationship between comorbidities and all-cause mortality, we established a logistic regression sequential model and gradually added the confounding variables to be adjusted. These confounding variables were identified by careful screening. We refer to previous high-quality papers in related fields, summarize the confounding factors corrected in these literatures, and adjust them as alternative solutions. After we have determined the confounders that need to be adjusted and alternative, we can make the final confirmation of alternative confounders through the results of univariate analysis. Variables with statistical significance (P < 0.05) were included in subsequent models for analysis. This study indicates that comorbidity in older people with hypertension increases the risk of all-cause mortality by 61–79%. In stratified analyses, the relationship between comorbidities and the risk of all-cause mortality persisted, particularly significant in females.

As the older people population increases, the number of patients with chronic diseases also increases [19]. Chronic disease coexistence refers to the coexistence of two or more chronic diseases in one individual, which is common in the older people over 60 years of age [20]. With the increase of age, the elasticity of the blood vessel wall decreases, and the body’s sensitivity to insulin hypoglycemia decreases. In addition, chronic diseases such as HTN and DM increase significantly under various unhealthy lifestyles, which plays a vital role in developing CI and poses a severe threat to human health [21, 22]. Studies have found potential links between high blood pressure and various chronic diseases [23]. HTN and DM are risk factors for CI [24]. The pathogenesis of HTN, DM, and CI is still unclear. On the one hand, it may be related to vascular damage caused by diseases, such as cerebral arteriosclerosis and cerebral energy metabolism disorders caused by HTN and DM, and cerebral microvascular lesions affect cognitive function to varying degrees. This chronic process eventually leads to CI [25, 26]. On the other hand, it may be related to vascular endothelial injury and impaired insulin conduction [27, 28].

Several studies have demonstrated an association between CI and an increased risk of all-cause mortality [29–33]. This study showed that HTN with CI had a significantly higher mortality risk. Multiple chronic diseases co-existing with HTN in the older people can exist independently and interact with each other, ultimately leading to the occurrence and development of CI. In the older people hypertensive population, it is suggested that preventing HTN complicated by multiple chronic vascular diseases and optimizing the management of HTN comorbidity are particularly important for protecting the cognitive function and prognosis of the older people hypertensive population.

In addition, this study found that the relationship between comorbidities and the risk of all-cause mortality in older female with hypertension appeared to be significant but not in male. We further calculated their effect size to be 0.11, indicating a small degree of difference. Previous studies have found that females had a higher prevalence of comorbidities than males [34, 35]. This phenomenon is usually associated with women being more sensitive to their own health [34], and may also be due to relatively few male cases in the study.

In 2012, the American Geriatric Society (AGS) proposed guidelines for managing comorbidity in the older people, which comprehensively address comorbidity from individuals willingness, clinical evidence, life expectancy, feasibility, and benefits of the program [36]. In 2017, the National Institute for Health and Care Excellence (NICE) issued guidelines for the clinical assessment and management of comorbidities in the older people, which manage comorbidities from the aspects of identifying risk groups, judging the impact of comorbidities, assessing frailty, managing multiple medications, and improving quality of life [37]. Therefore, how to better manage the older people with HTN comorbidity still needs more clinical research data.

The database used in this study was a nationally representative community-based cohort of the older people with HTN. Despite the difficulty of collecting data, few participants are lost to follow-up. Moreover, our study was prospectively designed and followed for ten years. The study has some limitations. Firstly, the proportion of men to women in the study was uneven, with fewer men than women. Future studies may need to expand the number of male participants. Secondly, the study only included participants who were aged ≥ 60 years people with hypertension. But on the other hand, this population was more susceptible to have comorbidities and easily to observe the trend happened, so as to take early prevention actions. Finally, comorbidities contain multiple diseases. This study only carries out systematic studies on HTN, DM, and CI. In the next step, comorbidities studies with larger sample size and more comprehensive prospective studies involving chronic diseases are required.

## Conclusion

The combination of DM and CI was associated with an increased risk of mortality in the older Chinese people with hypertension. In order to better manage the older people with HTN comorbidities, it is necessary to achieve comprehensive management suitable for the aged comorbidities in medical institutions of different levels through medical staff training and individualized continuous diagnosis and treatment.

## Data Availability

The research data used to support the finding of this study are available from the corresponding authors upon request.

## List of Abbreviations

CI: Cognitive impairment
DM: Diabetes mellitus
HTN: Hypertension
HRs: Hazard ratios
SBP: Systolic blood pressure
DBP: Diastolic blood pressure
MMSE: Mini-Mental State Examination
SD: Standard deviation
TC: Total cholesterol
HDL-C: High-density lipoprotein
LDL-C: Low-density lipoprotein cholesterol
TG: Triglycerides
SUA: Serum uric acid
Scr: Serum creatinine
CHD: Coronary heart disease
ADL: Activities of daily living
CCB: Calcium channel blockers
ACE: Angiotensin converting enzyme
ARB: Angiotensin receptor blocker
AGS: American Geriatric Society
NICE: National Institute for Health and Care Excellence
